# Pathogen‐binding nanoparticles to inhibit host cell infection by heparan sulfate and sialic acid dependent viruses and protozoan parasites

**DOI:** 10.1002/SMMD.20230046

**Published:** 2024-03-01

**Authors:** Adrian Najer

**Affiliations:** ^1^ Institute of Pharmaceutical Science King's College London London UK

**Keywords:** heparan sulfate proteoglycan (HSPG), metal nanoparticles, nanomedicines, parasite invasion, polymeric nanoparticles, sialic acid (SA), viral entry inhibitors

## Abstract

Global health faces an immense burden from infectious diseases caused by viruses and intracellular protozoan parasites such as the coronavirus disease (COVID‐19) and malaria, respectively. These pathogens propagate through the infection of human host cells. The first stage of this host cell infection mechanism is cell attachment, which typically involves interactions between the infectious agent and surface components on the host cell membranes, specifically heparan sulfate (HS) and/or sialic acid (SA). Hence, nanoparticles (NPs) which contain or mimic HS/SA that can directly bind to the pathogen surface and inhibit cell infection are emerging as potential candidates for an alternative anti‐infection therapeutic strategy. These NPs can be prepared from metals, soft matter (lipid, polymer, and dendrimer), DNA, and carbon‐based materials among others and can be designed to include aspects of multivalency, broad‐spectrum activity, biocidal mechanisms, and multifunctionality. This review provides an overview of such anti‐pathogen nanomedicines beyond drug delivery. Nanoscale inhibitors acting against viruses and obligate intracellular protozoan parasites are discussed. In the future, the availability of broadly applicable nanotherapeutics would allow early tackling of existing and upcoming viral diseases. Invasion inhibitory NPs could also provide urgently needed effective treatments for protozoan parasitic infections.

## Graphical abstract

Infections with viruses and protozoan parasites represent critical global health challenges. The search for effective broad‐spectrum applicable treatments for these diseases has sparked interest in developing nanotechnological therapeutic strategies. This mini‐review critically summarizes recent advances and future opportunities of nanoscale pathogen entry/invasion inhibitors, a specific class of anti‐infectious nanomedicines under development. (ToC schematic created with Servier Medical Art website CC‐BY).

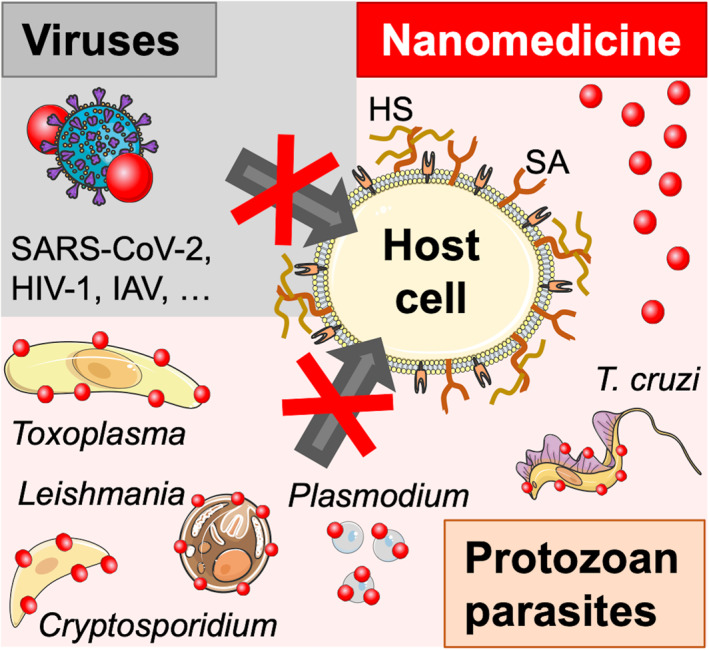

**Key points**

- Nanoparticles (NPs) that inhibit heparan sulfate (HS) and sialic acid (SA) dependent viruses and protozoan parasites through pathogen surface‐binding are discussed.
- For viral diseases, nanotechnological strategies with high inhibitory potency and virucidal mechanisms of action are highlighted.
- For protozoan parasites, invasion inhibition with soluble heparin and the few available NP‐based formulations are summarized to inspire more research into HS/SA‐mimetic NPs against these pathogens.

## 1 INTRODUCTION

Infectious diseases represent a major burden on global health. Among these, viruses and intracellular protozoan parasites are two types of pathogens responsible for devastating epidemics and pandemics. The COVID‐19 pandemic has highlighted that an emerging viral pathogen can rapidly become a huge threat to the global population, especially in the absence of broad‐spectrum antivirals. Indeed, for most viral infections there are no drugs available, which hinders effective control of current and future viral diseases. The development of broad‐spectrum applicable treatments would be particularly beneficial, as developing specific drugs for each viral disease would be very time‐consuming and costly. Broadly active antiviral medicines could help to (i) curb current viral infections, (ii) allow rapid response to infections with mutated strains that evade more specific strategies, such as vaccine‐induced antibodies, and (iii) ensure that upcoming diseases caused by new viruses can be counteracted quickly. With respect to (ii, iii), antiviral therapeutics could bridge the time needed for developing, testing, approving, manufacturing, and rolling out efficacious vaccines.

In contrast to viral diseases, protozoan parasitic diseases are caused by unicellular eukaryotic pathogens. Although this diverse group of pathogens also includes extracellular organisms (e.g., *Giardia lamblia*, *Trypanosoma brucei*, and *Entamoeba histolytica*), this review only focuses on parasites that require human host cells for propagation. These obligate intracellular protozoans include vector‐borne parasites (*Plasmodium* spp., *Trypanosoma cruzi*, and *Leishmania* spp.) and parasites distributed through the fecal–oral route (*Toxoplasma gondii* and *Cryptosporidium* spp.). Malaria caused by *Plasmodium* spp. is the most impactful protozoan disease. These parasites infect >200 million and kill >600,000 people every year, with children and pregnant women most severely affected.^1^ Malaria elimination programs are off track, with cases even increasing recently.^1^, ^2^ This highlights the need for innovation and development of alternative malaria control measures. Infection with *T. gondii*, sometimes referred to as malaria's neglected cousin, can lead to congenital toxoplasmosis, severe toxoplasmosis in immunocompromised people, and neuropsychiatric disorders.^3^ Leishmaniasis (*Leishmania* spp.) and Chagas disease (*T. cruzi*), categorized as neglected tropical diseases, are other parasitic infections affecting over 8 million individuals every year and they require urgent attention in terms of medicine development.^4^, ^5^, ^6^ Finally, *Cryptosporidium* spp. were recently found to be a major causative agent of diarrheal disease in children, registering >44.8 million episodes of diarrhea and 48,000 deaths annually.^7^, ^8^ For most protozoan parasite infections, no vaccines and only few, often ineffective treatments are currently available. The high prevalence and severity of infections caused by these different intracellular protozoan pathogens illustrates the urgent necessity to increase investment, develop new approaches, and conduct translational research to eventually reduce the high impact of these devastating diseases on global populations.

All human viruses and the obligate intracellular parasites discussed in this review utilize host cells for propagation. For viruses, the main entry pathways encompass ligand‐receptor interactions to allow subsequent fusion with the plasma and/or endolysosomal membrane. The mechanisms by which protozoan parasites enter host cells are extremely diverse and complex, multistep processes. They can mainly be categorized into parasite‐dominated invasion (*Plasmodium* spp., *T. gondii*, *Cryptosporidium* spp., and *T. cruzi*) and host cell‐mediated entry via phagocytosis (*Leishmania* spp.).^9^ Similar to the method used by viruses, host cell infection by parasites is initiated by pathogen ligand binding to host cell membrane receptors. Many specific host cell receptors have been identified for these different pathogens. However, in most cases, the pathogens only engage with these specific entry/invasion receptors after initiating binding via less specific, near universal attachment moieties. These shared initial attachment receptors are of high interest when developing broad‐spectrum anti‐infectious strategies targeting pathogen entry. Alternatively, pathogen‐tailored approaches can be designed by employing the more specific receptors/mimics. However, this will only achieve functionality against a single pathogen or pathogen type, which is not the focus of this review.

Glycans on host cell membranes represent these less specific universal attachment receptor moieties. These surface‐exposed polysaccharides that make up the glycocalyx are important for the initial interaction of most viruses and protozoan parasites with their respective host cell.^10^, ^11^, ^12^, ^13^ Highly anionic host heparan sulfate (HS) is one of these near universal interaction partners for pathogen ligands. Most viruses mentioned herein utilize HS interaction for host cell infection,^11^, ^14^, ^15^, ^16^ although in some cases it is still debated whether HS should be considered an actual receptor or not.^17^ HS on host cells is also involved in the invasion mechanism of all the five obligate intracellular protozoan parasites discussed herein.^11^, ^18^ A second broad‐spectrum example of a receptor moiety is sialic acid (SA). SA is part of many glycoproteins on host cell membranes and can mitigate viral and parasite entry.^10^, ^13^, ^19^ Consequentially, many anti‐infectious strategies targeting viral attachment/entry and parasite invasion have leveraged these HS/SA interactions.

Nanotechnology is gaining traction as a tool to develop alternative strategies for infectious diseases important in global health.^20^, ^21^ The highly successful lipid nanoparticle (LNP)‐based RNA vaccines for COVID‐19 have recently cemented the standing of nanotechnology as a promising and versatile engineering discipline for establishing infection prophylaxis. However, the potential of nanotechnology goes well beyond vaccines. Targeted delivery of antiviral agents^22^, ^23^, ^24^ and nanomedicines with intrinsic antiviral therapeutic properties, as discussed herein, are two other intervention strategies that could expand the viral control arsenal. The COVID‐19 pandemic has particularly sparked a huge interest in the development of broad‐spectrum therapies, which includes HS/SA‐inspired nanoparticles (NPs) as viral attachment/entry inhibitors.^25^, ^26^, ^27^ In contrast, the field of protozoan parasite invasion inhibitors based on NPs is still in its infancy. To date, most nanotechnological strategies against obligate intracellular protozoan pathogens have focused on vaccines, drug delivery, and direct toxic effects of mostly metal‐based NPs on parasites.^28^, ^29^, ^30^, ^31^, ^32^, ^33^, ^34^, ^35^, ^36^ However, the concept of invasion inhibition with NPs could provide important alternative therapeutic and/or prophylactic opportunities for protozoan parasites. The distinctively different mechanism of action of these NPs to current anti‐parasitic molecular drugs could represent a key advantage, especially in the context where drug‐resistant strains have developed and spread.

**FIGURE 1:**
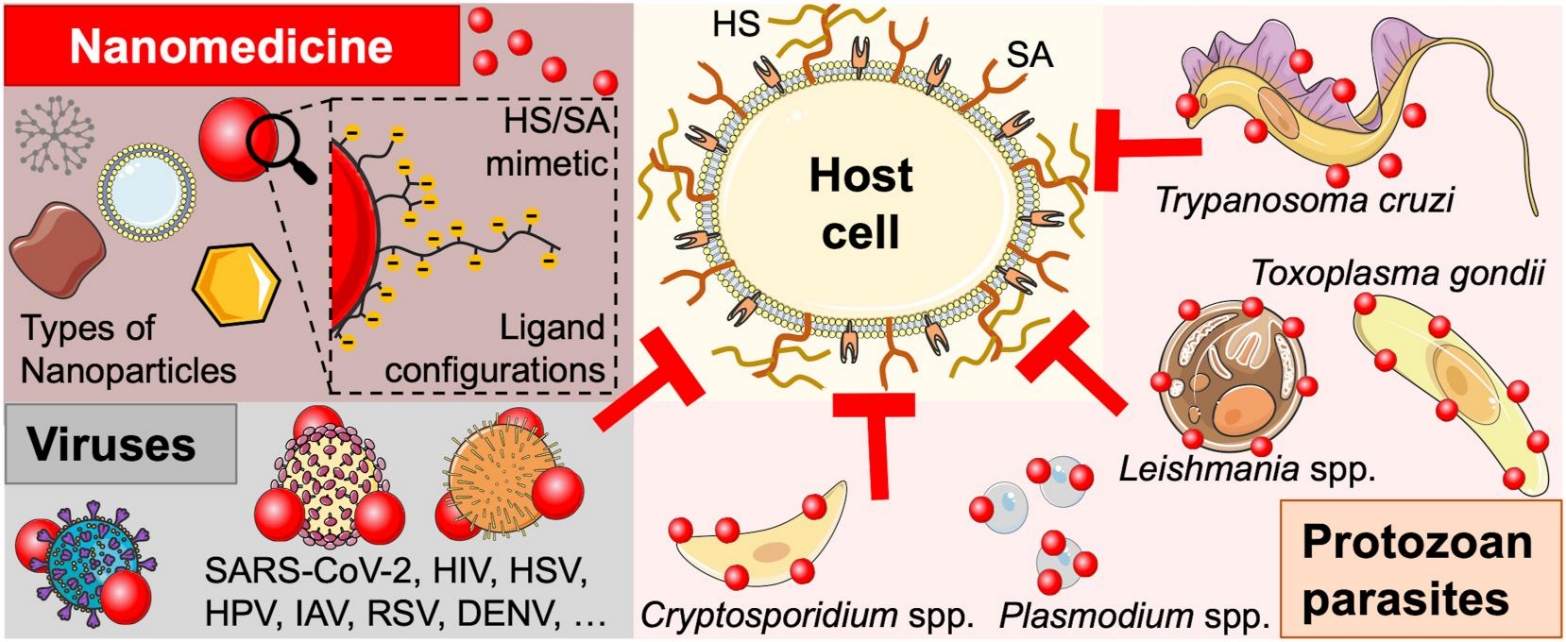
Schematic of virus and parasite entry/invasion inhibitory NPs. Nanomedicines are designed to mimic host HS and/or SA to inhibit pathogen attachment, entry, or invasion of host cells via direct pathogen surface binding. Possible ligand configurations on variously shaped NP types include short or long linkers, dendritic or polymeric groups in end‐on or side‐on attachment, all for the multivalent presentation of anionic sulfates/sulfonates or SA. Schematic created with Servier Medical Art website CC‐BY. DENV, Dengue virus; HIV, human immunodeficiency virus; HPV, human papillomavirus; HS, heparan sulfate; HSV, herpes simplex virus; IAV, influenza A virus; NPs, nanoparticles; RSV, respiratory syncytial virus; SA, sialic acid; SARS‐CoV‐2, severe acute respiratory syndrome coronavirus 2.

This review summarizes recent advances in the development of broad‐spectrum applicable viral attachment/entry and protozoan parasite invasion inhibitors based on NPs (Figure 1). First, antiviral strategies will be presented, splitting the information according to the NP type, from metal, soft matter, to other/non‐spherical NPs. Next, parasite invasion inhibitory NPs will be discussed in the context of malaria, as most NP research against protozoans up to now has focused on inhibiting *Plasmodium* spp. Nevertheless, the potential of NP strategies aimed at the extracellular forms of other parasites, such as *T. gondii*, *Cryptosporidium* spp., *T. cruzi*, and *Leishmania* spp., will then be discussed. Finally, a concluding section will put the recent findings into perspective to inspire more research in this direction.

## 2 BROAD‐SPECTRUM NP‐BASED VIRAL INHIBITORS

In the past, virus inhibitory NPs were mainly used as a research tool to identify new viral attachment and entry receptors.^37^ However, the overall rise of nanomedicine as a promising approach for various diseases has motivated the development of therapeutic antiviral NPs. The COVID‐19 pandemic, together with the high success of RNA‐loaded LNP coronavirus vaccines, provided an additional boost for the creation of therapeutic NPs for viral diseases. Here, some key examples and most recent works are highlighted rather than providing an exhaustive list of all the available nanostructures for viral entry inhibition. The reader is referred to other reviews on the topic for more breadth.^25^, ^26^, ^27^ Additional information on the history of polysaccharides and polymers for the same application, which set the basis for the NP developments, can also be found elsewhere.^25^, ^38^

Most broad‐spectrum applicable antiviral NPs have been designed by leveraging the HS^39^, ^40^, ^41^, ^42^, ^43^ and SA^19^, ^44^ interactions of viruses. This necessitates either using HS/heparin or SA as building blocks of NPs or mimicking these chemical entities by including sulfonates/sulfates or carboxylic acids in the NP design. Various possible ligand configurations have been trialed on different NP types and they include short or long linkers, dendritic or polymeric groups in end‐on or side‐on attachment, all with the aim of achieving multivalent presentation of anionic sulfates/sulfonates or SA (Figure 1). A key advantage of such anionic broad‐spectrum inhibitors is their potential to act against many different viruses. This design also provides higher robustness with respect to virus mutation. In contrast, more specific inhibitors, such as natural or vaccine‐induced neutralizing antibodies (NAbs), are prone to viral escape via mutation.^45^ However, the use of anionic inhibitors has historically been challenging in vivo, as found when aiming to translate polyanionic human immunodeficiency virus (HIV) inhibitors.^46^ Drawbacks of such inhibitors typically included low potency, reversibility of the interaction, and potential side effects (e.g., unwanted anticoagulation activity). These challenges required a re‐think of anionic NP designs. Recent advances in NP technology, as discussed in the next subsections, increased potency, included irreversible virucidal mechanisms and lowered the chance of side effects. These achievements have revitalized the research area of anionic NP‐based viral entry inhibitors (summarized in Table 1). Recent examples of HS/SA mimetic NP antiviral designs are discussed next by organizing them based on their core material.

**TABLE 1:** Efficacy comparison of selected antiviral NPs based on HS/SA mimetic structures, including a few historic examples but mostly recent formulations where EC_50_/IC_50_ values or curves were available.

NP type and reference	Size [nm]^a^	EC_50_/IC_50_ (virus type) [μg/mL]^b^	EC_50_/IC_50_ (virus type) [nM]^c^	Host cell	Mode of action	Animal model (virus)
(i) HS‐based						
Metal NPs						
AuNPs 1a‐Au^41^	1.7	1.3 (HIV‐1)	12 (HIV‐1)	MT‐2	n.a.^d^	‐
AuNPs MUS:OT^43^	2.8	10.9/1.6 (HSV‐1/2)	36.3/5.3 (HSV‐1/2)	Vero	Virucidal	Mice (RSV)
		4.1/9.2 (HPV‐16, RSV)	13.6/30.6 (HPV‐16, RSV)	293TT/A549	Virucidal	
^47^		1.4 (IAV)	6.3 (IAV)	MDCK	Virucidal	
^48^		5.4 (SARS‐CoV‐2)	17.0 (SARS‐CoV‐2)	VeroE6	Virustatic	
AuNPs L2‐3^49^	77	4.3 (DENV)	‐	Vero	Virucidal	‐
AuNPs PSS^50^	5–40	>0.1 (HIV‐1, RSV)^e^	‐	TZM‐bl/A549	Virustatic	Mice (RSV, for SARS‐CoV‐2, PSS only)
		∼10 (HSV‐1, ZIKV)		ELVIS/VeroE6	Virustatic	
		>100 (SARS‐CoV‐2)		Caco‐2	Virustatic	
Soft matter NPs						
Liposome‐Hep^51^	88	0.061 (HSV‐1, RSV)	0.0004 (HSV‐1, RSV)	Vero	n.a.	‐
SulfoLiposome^52^		120 (SARS‐CoV‐2)	‐	Vero	Virucidal	‐
Nanogel F‐NG^53^	212	∼90 (HSV‐1)	‐	VeroE6	Virustatic	‐
Dendrimer‐ dPG5^54^	5	0.16 (HSV‐2)	4.5 (HSV‐2)	VeroE6	Virucidal	‐
		0.15 (RSV)	4.2 (RSV)	A549	Virucidal	‐
Nanomimics AMSA^55^	35	0.00001 (HSV‐2)	0.000006 (HSV‐2)	Vero	Virustatic	‐
		19 (SARS‐CoV‐2)	15 (SARS‐CoV‐2)	Vero	Virustatic	
Other NPs						
Hexagonal OPH1‐Hep4^56^	660	0.86/0.42 (HSV‐1/2)	‐	Vero	n.a.	‐
		2.2/1.0 (HPV‐16/RSV)		293TT/A549		
Graphene‐PGS^57^	∼100	0.8 (SARS‐CoV‐2)	‐	VeroE6	Virucidal	‐
Fullerene‐IPGS^58^	∼100	∼10 (SARS‐CoV‐2)	‐	VeroE6	n.a.	‐
Alg@AS_5.0_ ^59^	150	∼250 (IAV)	‐	MDCK	n.a.	Mice (IAV)
(ii) SA‐based						
Nanogel‐SA^60^	257	23 (IAV)	0.0023 (IAV)	MDCK‐II	n.a.	‐
PAMAM‐SA^61^	4.5	‐	170 (IAV)	MDCK	n.a.	Mice (IAV)
Phage NP SA^62^	25	‐	0.04 (IAV)	A549/MDCK‐II	n.a.	Mice (IAV)
Fil‐phage SA^63^	1000	‐	0.014 (IAV)	MDCK	n.a.	‐

Abbreviations: AMSA, aminomethanesulfonic acid; DENV, Dengue virus; HIV, human immunodeficiency virus; HPV, human papillomavirus; HS, heparan sulfate; HSV, herpes simplex virus; IAV, influenza A virus; NPs, nanoparticles; PGS, polyglycerol sulfates; PSS, polystyrene sulfonate; RSV, respiratory syncytial virus; SA, sialic acid; SARS‐CoV‐2, severe acute respiratory syndrome coronavirus 2; ZIKV, Zika virus.

^a^ Hydrodynamic or core diameter given.

^b^ >/∼ are used when no numbers were available but the values were estimated from the EC_50_/IC_50_ curves.

^c^ Refers to NP concentration.

^d^ n.a. refers to “not available,” because no information on the mechanism was available.

^e^ See Figure 3D for a graphical representation.

### 2.1 Metal NP‐based viral inhibitors

Antiviral applications of metal NPs, such as Au‐ and Ag‐based systems, have recently been reviewed extensively.^64^, ^65^, ^66^, ^67^ Here, selected highlights that function by direct virus binding are summarized. These examples are then compared to antiviral NPs made from other materials. In early studies, the multivalency of HS‐inspired metal NPs was already identified as the key factor for virus entry inhibition. This is exemplified by studies from Baram‐Pinto et al. who demonstrated that soluble monovalent mercaptoethanesulfonate (MES) did not affect herpes simplex virus type 1 (HSV‐1) host cell entry, while multivalent MES‐coated silver and gold NPs (AgNPs and AuNPs) reduced infectivity.^39^, ^40^ This same effect was found when testing sulfated AuNPs against human immunodeficiency virus 1 (HIV‐1).^41^ Analogously, changing to multivalent SA‐modified AuNPs delivered inhibitors for SA‐dependent viruses such as influenza A virus (IAV).^68^

Another early exploration was around optimizing the NP size for inhibition. Many of the viruses discussed herein are in the size range of around 100 nm in diameter. This length scale is comparable to nanomedicines, highlighting the importance of inhibitor size. Vonnemann et al. revealed that polyvalent virus‐sized AuNPs were the most efficient attachment inhibitors compared to smaller or bigger NPs when tested against a model pathogen, vesicular stomatitis virus.^42^ The virus‐sized NPs provided increased activity due to better cross‐linking of several virions versus smaller NPs. However, when considering the NP size, the binding strength has to be included in these considerations.^69^ In addition, the optimal inhibitor size might be virus specific, while other mechanisms of inhibition, for example, damaging the virus irreversibly as discussed below, could require a different optimal NP size.

**FIGURE 2:**
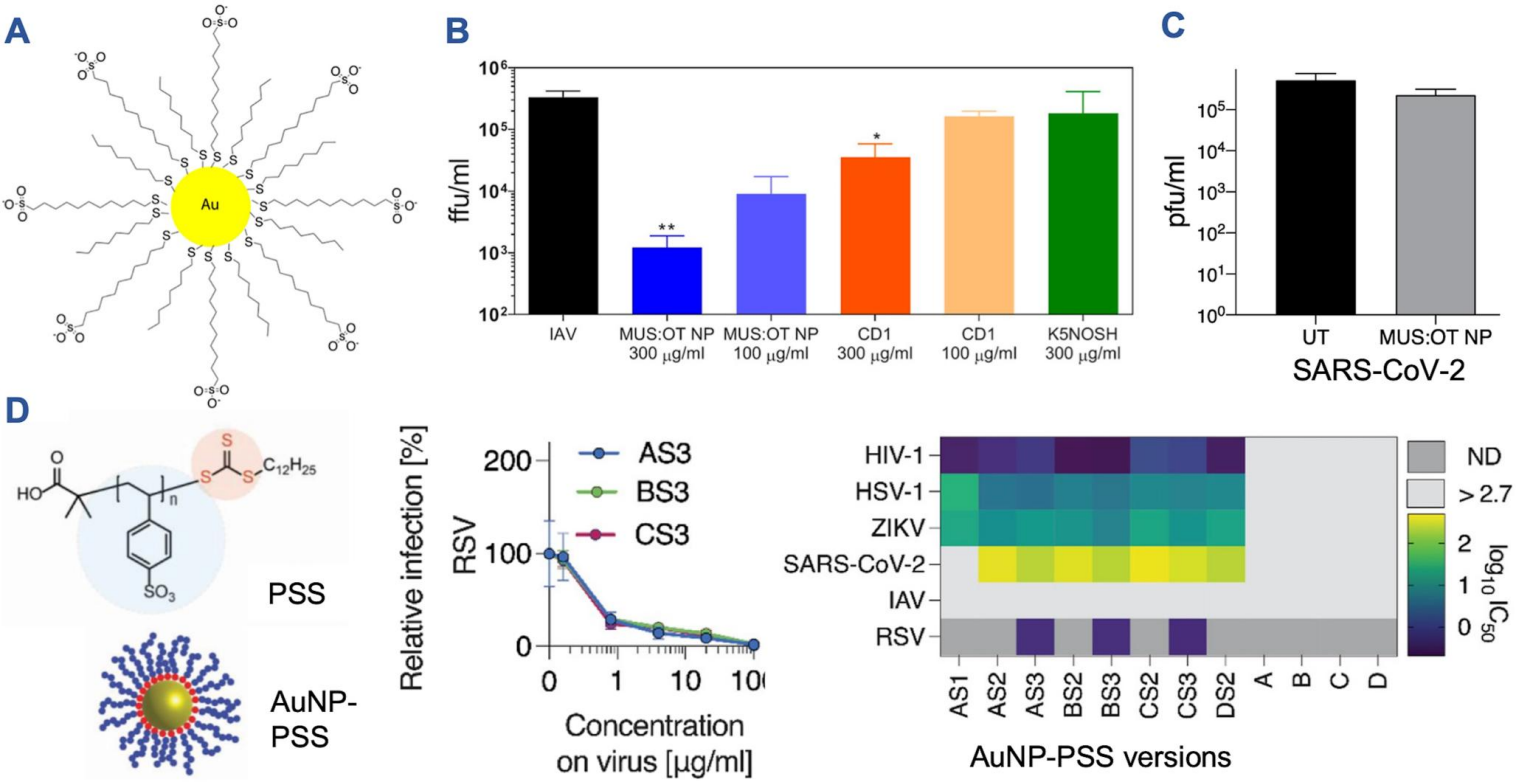
Antiviral gold nanoparticles. (A, B) MUS:OT AuNP schematic and virucidal activity against IAV compared to other inhibitors. *p < 0.05, **p < 0.01. Modified and reprinted with permission.^47^ Copyright 2020, American Society for Microbiology. (C) Virucidal activity test of MUS:OT AuNPs against SARS‐CoV‐2, revealing no virucidal activity but only virustatic activity. Modified and reprinted under terms of the CC‐BY license.^48^ Copyright 2020, The Authors, published by MDPI. (D) Schematic of AuNP‐PSS, IC_50_ example curves for virustatic activity against RSV, and illustration of IC_50_ for various AuNP sizes and PSS molecular weights when tested against different viruses. Modified and reprinted under terms of the CC‐BY license.^50^ Copyright 2022, The Authors, published by John Wiley and Sons. IAV, influenza A virus; PSS, polystyrene sulfonate.

More recently, Cagno et al. have made a significant contribution to the field of antivirals by incorporating a virucidal mechanism into HS‐mimetic AuNPs.^43^ This was achieved by including long hydrophobic linkers between the Au core and the sulfonate functional groups (Figure 2A). These inhibitors were tested under in vitro conditions that better mimic the in vivo situation where the inhibitor will eventually be diluted over time. They found a potent virucidal effect for their mercaptoundecanesulfonic acid/octanethiol (MUS/MUS:OT)‐AuNPs against various viruses, including herpes simplex virus type 2 (HSV‐2), human papillomavirus, respiratory syncytial virus (RSV), and Dengue virus (DENV). The activity was confirmed in a mouse RSV model. In contrast, the MES‐AuNPs introduced above contained much shorter linkers and provided only virustatic activity. The virucidal mechanism for MUS/MUS:OT‐AuNPs was characterized by electron microscopy and revealed virus binding and irreversible viral deformation. This functionality was attributed to the strong multivalent interactions of MUS/MUS:OT‐AuNPs with the virus, mediated through the sulfonate groups together with the long hydrophobic linkers. Changing the sulfonated MUS component on these virucidal AuNPs to a multi‐sulfonated complex ligand based on glucose yielded slightly improved virucidal activity against DENV.^49^ Intriguingly, when exploring the activity of MUS:OT‐AuNPs against other viruses that do not use HS interaction for host cell attachment (e.g., IAV), they were still functional (Figure 2B).^47^ However, Cagno et al. revealed recently that MUS:OT‐AuNPs only provided virustatic and not virucidal activity against severe acute respiratory syndrome coronavirus 2 (SARS‐CoV‐2, Figure 2C), the causative agent of the COVID‐19 pandemic.^48^ Although HS was previously identified as an attachment receptor for SARS‐CoV‐2,^16^ others have found no inhibition with heparin^48^ or only at very high mg/mL concentration.^55^ This could point toward an explanation for the as of yet unexplained absence of a virucidal activity of MUS:OT‐AuNPs against SARS‐CoV‐2. More research is required to better understand SARS‐CoV‐2 inhibition with sulfonated/sulfated compounds and to turn these NPs virucidal against this specific virus.

Protein corona formation on surface‐active NPs, same as for all other nanomedicines,^70^, ^71^ is one underexplored but highly important aspect toward future clinical translation. One attempt to address this challenge modulated virucidal MUS‐AuNPs from anionic to mixed charge to increase their potency in high protein environments.^72^ Since the virucidal activity of the above MUS/MUS:OT‐AuNPs still required relatively high concentrations of inhibitor, also when tested in the virustatic assays,^43^ it is important to identify more potent structures. Many groups have been evaluating other types of modifications on AuNPs to achieve higher virustatic activity. Avoiding the hydrophobic linkers could potentially be associated with lower toxicity.^50^ Conversely, incorporating these long‐chain linkers might turn more potent virustatic NPs virucidal. Groß et al. have recently explored the formulation of polystyrene sulfonate (PSS)‐coated AuNPs against various viruses (Figure 2D).^50^ These PSS‐AuNPs revealed virustatic activity against many viruses (except IAV that does not rely on HS), while SARS‐CoV‐2 was again the most challenging to inhibit. Despite these challenges, the activity was confirmed in mouse models of RSV and SARS‐CoV‐2 infection, respectively.

Many other metal‐based NPs possess antiviral properties as reviewed elsewhere.^64^, ^65^, ^66^, ^67^ However, it is often difficult to distinguish between different inhibition mechanisms. For example, direct NP action on the virus or indirect NP activity mediated via induction of host cell changes cannot always be decoupled. A recent study that highlights the issue of interconnected multifunctionality used Au nanoclusters with terminal ammonium groups (TMA‐GNCs).^73^ The inhibition mechanism was found to be virucidal but functioning through multi‐fold mechanisms by concurrently attacking and destroying the virus itself, reducing coronavirus protease 3CL^pro^ activity, and activating antiviral immune responses. Similarly, other metal‐based NP types can have some effect on viral attachment/entry, but it is often combined with other effects on host cells. A common phenomenon is strong stimulation of the interferon pathway by metal NPs in the host cells.^64^, ^74^ This can result in an indirect antiviral effect as exemplified by studies developing antiviral graphene oxide‐AgNP composites and glutathione‐capped silver sulfide nanoclusters.^75^, ^76^ While this might be of benefit to reduce viral load, it might also cause unwanted side effects. Overstimulation of the host immune system could have a detrimental effect and must be evaluated very carefully.^64^ Although metal‐based NPs have shown potential as viral inhibitors, persistent concerns over potential toxic side effects caused by some of these NPs are reasons for caution.^77^ This is also why more biocompatible and biodegradable systems, such as soft matter‐based NPs, are being sought after.

### 2.2 Soft matter‐based nanoscale viral inhibitors

Soft matter‐based NPs, made from lipidic, polymeric, and dendritic components, have unique potential for anti‐infectious applications. High biocompatibility and the potential to include biodegradable components for many of these NPs is a key advantage over other NP types. Their often characteristic softness also allows NP deformation upon virus binding. This deformation can increase the number of interaction points between NP and virus, which enhances binding strength, and ultimately improves the inhibition potential.^53^ Arguably the most researched soft matter‐based NPs are liposomes, which are vesicles composed of a lipidic membrane and an aqueous core. For example, heparin octasaccharide‐modified liposomes successfully inhibited viral attachment and host cell infection by RSV and HSV‐1.^51^ Using only heparin fragments (octasaccharide) ensured the removal of the unwanted anticoagulation activity characteristic for full‐length heparin. Instead of oligomeric heparin fragments on liposomes, sulfated liposomes were created by incorporating cholesteryl sodium sulfate,^52^ which achieved SARS‐CoV‐2 inhibition and inactivation, partially through fusion with virions, although much higher liposome concentrations were necessary compared to heparin octasaccharide‐modified liposomes (Table 1). Other glycan‐modified liposomes with pendant SA groups were also developed to function against IAV, with activity confirmed in mice with lethal IAV infection.^78^, ^79^ Liposomes that mimic natural membranes have the advantage of allowing lateral mobility of the inhibitory receptor/mimic. This configuration is more biomimetic and can be one explanation for the high potency of the heparin octasaccharide‐modified liposomes versus other NPs (Table 1).^51^ High biocompatibility of liposomes due to their membrane‐mimetic design using natural lipids is another key advantage over other NP systems. As one drawback, liposomes are often associated with limited physicochemical stability, which needs to be considered when moving toward the translation. Especially, taking into account the various local environments encountered by NPs depending on the chosen administration route is important. Using heparin as a NP building block was also explored in the development of an inhalable solution of polyplexes based on chitosan‐heparin mixtures, which achieved activity in a mouse model with SARS‐CoV‐2 infection.^80^ However, the use of full‐length heparin in the construction of these NPs has to be evaluated very carefully with respect to potential downstream anticoagulation side effects once the NPs start to disassemble.

**FIGURE 3:**
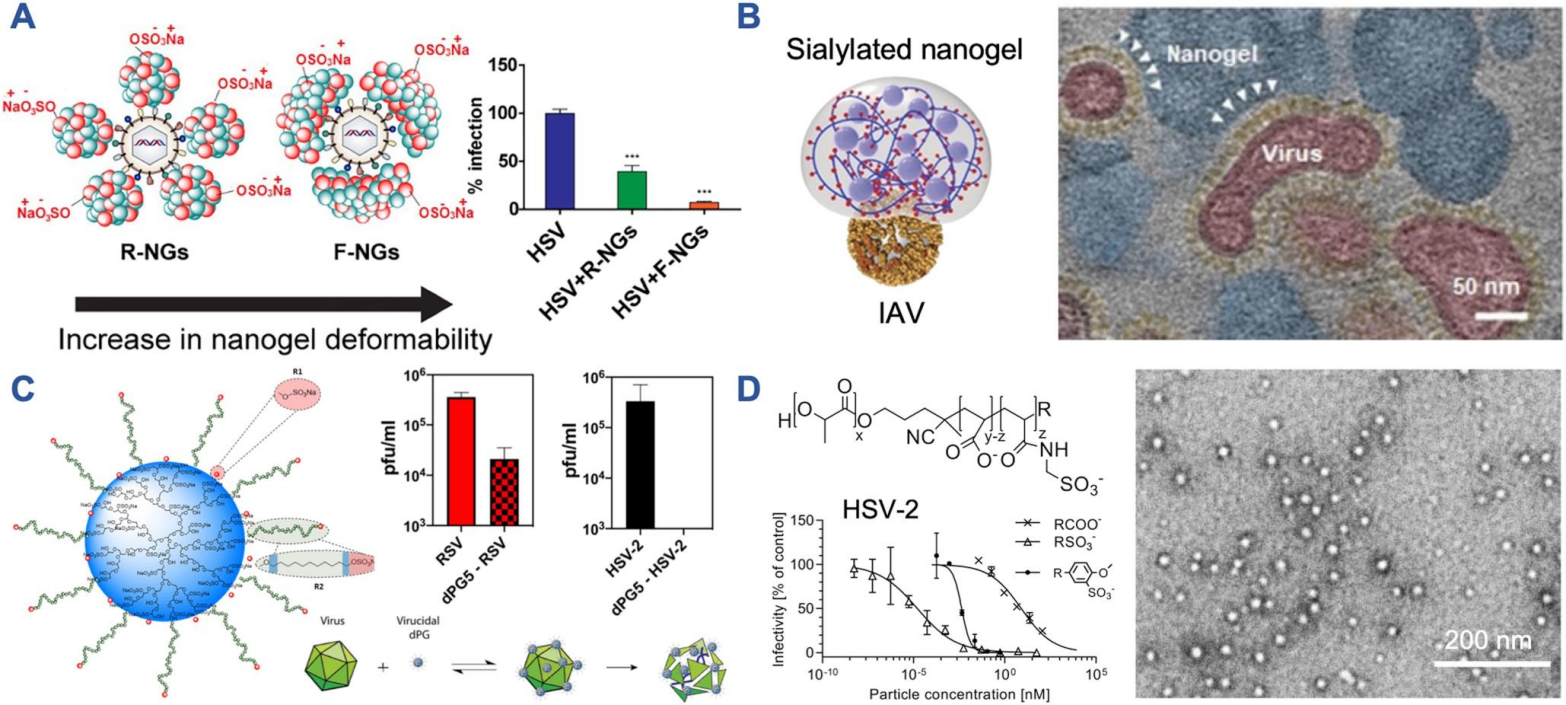
Soft matter‐based antiviral nanoparticles. (A) Sulfated NGs with increased deformability (F‐NGs) inhibited HSV‐1 infection of host cells more efficiently than more rigid structures (R‐NGs). ***p < 0.001. Modified and reprinted with permission.^53^ Copyright 2018, American Chemical Society. (B) SA‐modified NGs were also active against IAV, again revealing deformation of the NGs upon virus binding as shown in this artificially colored electron micrograph. Modified and reprinted under terms of the CC‐BY license.^60^ Copyright 2020, The Authors, published by John Wiley and Sons. (C) A virucidal mechanism against RSV and HSV‐2 could be incorporated into a sulfonated soft dendritic NP system by including long hydrophobic linkers. Modified and reprinted with permission.^54^ Copyright 2022, American Chemical Society. (D) Degradable micelles (electron micrograph on the right) made of a copolymer that incorporates a random carboxylated/sulfonated block (${\text{RSO}}_{3}^{‐}$ sample) were found to be highly potent virustatic inhibitors of HSV‐2 (IC_50_ in the fM region). Modified and reprinted under terms of the CC‐BY license.^55^ Copyright 2022, The Authors, published by American Chemical Society. HSV‐2, herpes simplex virus type 2; IAV, influenza A virus; NGs, nanogels; RSV, respiratory syncytial virus; SA, sialic acid.

Most other soft matter‐based NP systems for viral inhibition are based on heparin‐mimetic structures rather than using heparin. Alternatively, SA units are incorporated for SA‐dependent viruses. Nanogels (NGs) with sulfate end groups revealed the benefit of using more flexible versus rigid viral binding structures. HSV‐1 was inhibited more efficiently when the NGs were more flexible (Figure 3A).^53^ When modifying the NGs with SA instead, flattening of the flexible NGs when binding to IAV virions was confirmed by electron microscopy (Figure 3B).^60^ Again, an inhibitor size similar to the virus was ideal for inhibition, while the ligand density needed to be optimized carefully.^81^ Utilizing a dendritic system, Kwon et al. showed precisely how the inter‐ligand spacing is an important factor determining the activity of SA‐NPs against IAV.^61^ Their lead dendrimer formulation (S3‐G4, 3.1 nm ligand spacing) was the most potent and applicability was confirmed in a lethal influenza mouse model. The necessity of choosing the correct type of SA on NPs was realized through findings that avian and human IAV were inhibited better with dendrimers containing 3′‐sialyllactose (3SL) or 6′‐sialyllactose (6SL), respectively.^82^

In attempts to incorporate a virucidal inhibition mechanism into more readily translatable, soft backbone structures, cyclodextrins were evaluated.^83^, ^84^ Again, long hydrophobic linkers with sulfonate (MUS) or SA end groups were incorporated. Virucidal action was achieved for these modified cyclodextrins against a whole range of viruses (HSV‐1/2, RSV, HIV‐1, DENV2, Zika virus, and hepatitis C virus); however, the overall potency was lower than for the AuNP core structures.^43^ In the search for higher potency soft matter‐based virucidal structures, the MUS modification was recently transferred to a dendritic system.^54^ This design created the highest potency virucidal HS‐mimetic NPs to date (Table 1, Figure 3C). However, whether the in vitro data directly translate to an in vivo situation has not yet been demonstrated for this specific structure. Switching to micellar polymeric NP systems for viral inhibition, Najer et al. have recently demonstrated that modulating the particle surface chemistry from carboxylates to a mixture with sulfonates can produce highly potent virustatic inhibitors of HSV‐2 (Figure 3D).^55^ The IC_50_ values were in the femtomolar region (pg/mL) for the lead NP (aminomethanesulfonic acid [AMSA]‐modified poly(*D,L*‐lactide)‐*block*‐poly(acrylic acid) (PDLLA‐*b*‐PAA) copolymer micelles). The reasons for this high inhibitory potential against HSV‐2 are yet unknown. Evaluation of binding affinities, multivalency, and potential activation of host responses, as described in the metal NP part, are potential future works to establish the mechanism. As one caveat, when testing the same inhibitors against SARS‐CoV‐2 they were much less potent.^55^ The difficulty of obtaining inhibition with heparin in these latter assays (required mg/mL) represents one possible explanation for this discrepancy.

Besides broad‐spectrum applicable soft matter‐based NPs, there is also a lot of interest and research ongoing in developing NP versions that include specific receptors. For more details on these inhibitors, which were mostly excluded from this review, the reader is referred to cited papers below and a recent review specific to NPs including ACE2 engineering for SARS‐CoV‐2.^85^ Recent specific examples include peptide‐modified NPs (IAV),^86^ NPs based on molecularly imprinted polymers (MIPs against HIV‐1, SARS‐CoV‐2),^87^ receptor presenting virus‐like NPs (HIV‐1),^88^ liposomes (SARS‐CoV‐2),^89^ cell‐membrane vesicles (SARS‐CoV‐2,^90^, ^91^, ^92^, ^93^, ^94^, ^95^ hepatitis B virus,^96^ HIV‐1,^95^ IAV,^97^, ^98^ HSV‐1,2 and pseudorabies virus^99^), and extracellular vesicles (SARS‐CoV‐2).^100^ This field has progressed up to successful trials in non‐human primates using the cell‐membrane‐based nanodecoys presenting ACE2 against SARS‐CoV‐2.^92^, ^94^ Most of the NP types discussed up to now were based on a spherical morphology. Recent progress in the design of 2D materials and more complex 3D architectures has given rise to other classes of nanomaterials being investigated for viral attachment/entry inhibition.

### 2.3 Other nanomaterials for virus inhibition

Employing natural base components, such as bacteriophages, generally produces more morphologically defined structures compared to most human engineered NPs. Monodisperse phage capsids functionalized with SA groups enabled studies on the impact of precise ligand spacing on NPs for IAV inhibition.^62^, ^63^ Icosahedral bacteriophages Qβ, with SA spacing optimized to fit the distance between individual binding sites on the hemagglutinin trimer (∼4.7 nm), potently blocked IAV in vitro, ex vivo, and in vivo.^62^ Others showed that sialyllactose‐conjugated filamentous bacteriophages (∼1 μm long) can wrap around IAV to yield high inhibition potential, with the lowest IC_50_ at 14 pM.^63^

**FIGURE 4:**
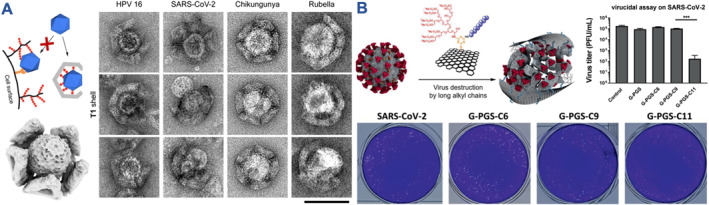
Non‐spherical nanodesigns for virus inhibition. (A) Schematic of heparin/HS modified T1 DNA origami shells and corresponding electron micrographs showing successful caging of various virus types (Scale bar, 100 nm). Modified and reprinted under terms of the CC‐BY license.^106^ Copyright 2022, The Authors, published by American Chemical Society. (B) 2D graphene nanosheets with sulfated dendritic groups and hydrophobic chains (≥10 carbons in length) achieved potent SARS‐CoV‐2 inhibition with a virucidal mechanism (bottom images show plaque assay). ***p < 0.001. Modified and reprinted under terms of the CC‐BY license.^57^ Copyright 2021, The Authors, published by John Wiley and Sons. HS, heparan sulfate.

Instead of binding NPs to the surface of viruses to protect host cells from infection, so‐called virus‐traps were established. These traps are defined as nanoscale constructs with cavities to bind the virus inside their pockets. DNA nanotechnology is particularly suited for such designs as it allows precise engineering of 2D‐ and 3D‐architectures. This advantage has recently been leveraged to develop anti‐pathogenic strategies, including against viral entry.^101^ It was shown that incorporating viral target‐specific peptides^102^ or aptamers^103^, ^104^, ^105^ into these DNA nanoconstructs produces specific inhibitors. One broad‐spectrum option employing very defined viral traps was designed by Monferrer et al. using DNA origami technology (Figure 4A).^106^, ^107^ Heparin or HS was immobilized on the inside of these DNA origami shells to encage various types of viruses. The mode of action was proposed to be the same as for other NP‐based inhibitors, subsequently preventing interaction of the virus with host cell membranes. Keeping the virus within the trap was proposed to passivate the virus surface better than soluble inhibitors (e.g., free HS). However, efficacy data on reducing virus infectivity, comparing this system to soluble inhibitors and other NP types, has not yet been presented.

Carbon structures such as graphene, carbon dots, nanotubes, and fullerenes have also been explored as antivirals.^108^, ^109^ Some of these structures have intrinsic antiviral activity or they were modified with non‐HS/SA mimetic moieties, such as mannose for Ebola virus inhibition,^64^ which are not further discussed herein. In turn, broad‐spectrum applicable HS‐mimetic sulfonated/sulfated graphene inhibited host cell attachment of HSV‐1, pseudorabies, and African swine fever virus.^110^, ^111^ Hydrophobic chains (≥10 carbons in length) were again included and combined with sulfated dendritic groups on flexible 2D nanomaterials such as graphene (Figure 4B).^57^, ^112^ This yielded potent activity and a virucidal mechanism of action against HSV‐1 and SARS‐CoV‐2. However, the increased hydrophobicity was also associated with higher toxicity in host cells. Due to the high inhibitory potential with IC_50_ in the fM range when tested against HSV‐1, application at low concentrations below the toxicity limit could be explored.^112^ The inhibitory potential is much higher than for most other NP types and similar to the highly potent virustatic AMSA‐modified polymer micelles functioning against HSV‐2.^55^ Hence, further investigations into mechanism are warranted to push these highly potent inhibitors closer to clinical translation. The same 2D graphene platform was also effective against IAV by substituting the sulfates with SA moieties.^113^ Another two examples highlight the benefit of combining the hydrophobicity of carbon materials with sulfates for viral inhibition. Firstly, the intrinsic hydrophobicity of fullerene (buckyballs) was combined with anionic charge (polyglycerol sulfates) to block SARS‐CoV‐2.^58^ Secondly, a carbonized NG with sulfate surface functionalities (Alg@AS_5.0_) was active against IAV, including in an in vivo influenza model.^59^ Again, the activity of this sulfated carbonized NG against IAV that does not rely on HS for host cell attachment might broaden the applicability of this material, as it was the case for the MUS:OT AuNPs.^47^

Other 2D nanocomposites were made from sulfonated transition metal carbides (Ti_3_C_2_‐Au‐MPS) with activity demonstrated against SARS‐CoV‐2 pseudovirus but only at relatively high concentrations (lowest 50 μg/mL).^114^ A recent report on electronegative 2D CuInP_2_S_6_ (CIPS) nanosheets revealed strong antiviral activity against SARS‐CoV‐2 (EC_50_ at 11 pM), which was retained in vivo.^115^ The high activity was attributed to the strong affinity of the nanosheets to the receptor binding domain of the spike protein (*K*_*D*_ < 1 pM). Another NP type with non‐spherical shape, hexagonal nanoassemblies made from *O*‐palmitoyl‐heparin and α‐cyclodextrin (OPH1‐Hep4) efficiently inhibited a range of HS‐dependent viruses, with a high degree of sulfation required to achieve high efficiency.^56^ Overall, non‐spherical shapes that match the target virion or flexible 2D‐ or 3D‐inhibitory structures that can wrap around the virions are upcoming materials with high potential. However, direct comparison to other inhibitory materials is still sparce. Hence, a definitive conclusion on whether they are advantageous over spherical inhibitors remains to be drawn. The focus of this review is now shifted away from viruses to protozoan parasites that utilize the same HS/SA interactions for interaction with host cells. Theoretically, the same NPs described in the preceding sections can be applied against parasites. However, as discussed subsequently, differences in parasite versus virus biology and variations in entry mechanisms requires slightly different anti‐parasitic designs for optimal activity.

## 3 NPs TO INHIBIT PROTOZOAN PARASITE INVASION OF HOST CELLS

Most anti‐parasitic nanomedical strategies have previously explored drug delivery applications and inherent toxic effects of predominantly metal‐based NPs on parasites.^28^, ^29^, ^30^, ^31^, ^32^, ^33^, ^34^, ^35^, ^36^ Herein, these examples are not discussed in detail. Instead, the focus of this review is on broad‐spectrum NPs that function as invasion inhibitors by occupying parasite surface ligands. *Plasmodium* spp., the unicellular organism causing malaria, represents the most impactful protozoan parasite in global health. Hence, most of the nanotherapeutic anti‐invasion strategies have been aimed at this parasite to date. A summary of these approaches is given herein. Future work is then proposed to tackle other protozoan parasites with NPs employing the same mechanism of action.

### 3.1 Malaria parasite invasion inhibition with NPs

Already several decades ago, liposomes with specific host receptors were evaluated as invasion inhibitors of *Plasmodium* merozoites, with a focus on identifying parasite receptors rather than designing therapeutics, as seen in the virus field.^116^, ^117^ As the production of NPs, including liposomes, has become much more economical throughout the last decades, the interest in developing NP‐based invasion inhibitors has gained more interest. Particularly, designs with low‐cost, broad‐spectrum applicable receptors, such as HS, or simple receptor mimics are prime candidates to develop anti‐parasitic NPs. Encouragingly, red blood cell (RBC)‐infecting *Plasmodium* spp. merozoites use HS interaction to invade RBCs and the process can be inhibited with soluble heparin.^118^ Heparin‐like molecules have since been investigated broadly for *Plasmodium* invasion inhibition.^118^, ^119^, ^120^, ^121^ However, only a few NP types with heparin or heparin‐mimetic surfaces have so far been developed and tested for an invasion‐inhibitory application (Table 2).

**TABLE 2:** Efficacy comparison of malaria parasite invasion inhibitory NPs based on HS.

NP type	Structure	Size [nm]	EC_50_/IC_50_ (strain) [μg/mL]^a^ ^,^ ^b^	EC_50_/IC_50_ (strain) [nM]^b^ ^,^ ^c^	Animal	Refs
Lipo‐Hep‐1	Liposome (covalent side‐on)^d^	181	∼1 (3D7)	‐	‐	122
Lipo‐Hep‐2	Liposome (covalent side‐on)	∼200	∼10 (3D7)	‐	Mice (circulation)	123
Chitosan/Hep	Polyplex (electrostatic)	140	∼3 (3D7)	‐	‐	122
DHP(G4)‐MPA‐Hep	Dendrimer (electrostatic side‐on)	∼70	4.3 (3D7)	‐	‐	124
Nanomimics‐Hep	Polymersome (covalent end‐on)	132	0.2 (3D7)	0.3 (3D7)	‐	125, 126
HMFN@Hep	Inorganic NP (electrostatic side‐on)	196	7.9 (3D7)	‐	‐	127
CarboxyNP‐PAA	Polymer micelle (covalent end‐on)	35	2.5/2.5 (3D7/D10)	4.0/4.1 (3D7/D10)	‐	55
			1.1 (W2mef)	1.8 (W2mef)		
			0.4 (A1‐H.1)^e^	0.7 (A1‐H.1)		
SulfoNP‐AMSA		35	3.8/5.0 (3D7/D10)	2.9/3.8 (3D7/D10)		
			2.3 (W2mef)	1.8 (W2mef)		
			0.4 (A1‐H.1)	0.3 (A1‐H.1)		
SulfoNP‐AMBS		35	2.0/1.6 (3D7/D10)	0.9/0.7 (3D7/D10)	Mice (*Plasmodium berghei*)	
			1.7 (W2mef)	0.7 (W2mef)		
			1.7 (A1‐H.1)	0.7 (A1‐H.1)		
AuNP MUS:OT	Gold nanoparticle	2.8	∼40 (3D7)	‐		
PLN‐AMBS‐PEG	Polymer/lipid vesicle (covalent end‐on)	∼100	0.9 (3D7)	0.4 (3D7)	Mice (*P. berghei*)	55

Abbreviations: AMSA, aminomethanesulfonic acid; HS, heparan sulfate; NPs, nanoparticles; RBC, red blood cell.

^a^ Concentration of inhibitory component.

^b^ Host cells for in vitro testing were human RBCs.

^c^ Refers to NP concentration.

^d^ Inhibitory polymer conjugation and orientation.

^e^ *Plasmodium knowlesi* strain A1‐H.1 adapted to human RBCs.^128^

**FIGURE 5:**
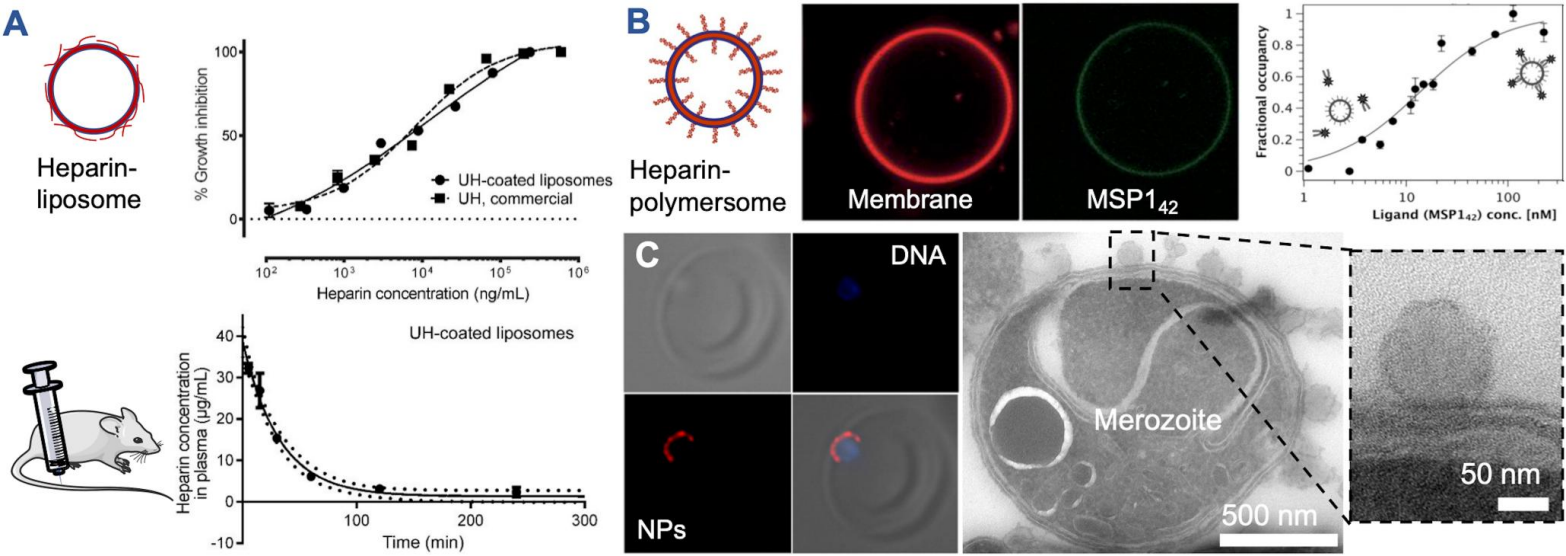
*Plasmodium* merozoite invasion inhibition with heparin‐nanovesicles. (A) Heparin‐coated liposomes (side‐on configuration) inhibited merozoite invasion but showed relatively short circulation times. Modified and reprinted under terms of the CC‐BY license.^123^ Copyright 2020, The Authors, published by MDPI. Schematic modified from Servier Medical Art website CC‐BY. (B) Giant heparin‐polymersomes (ca. 15 μm in diameter, red membrane stain) bound fluorescent *Plasmodium falciparum* parasite ligand *Pf*MSP1_42_‐OG488 (green). Modified and reprinted with permission.^130^ Copyright 2016, Swiss Chemical Society. Nanoscale versions of the same polymersomes revealed high binding strength for this interaction (*K*_*D*_ ∼12 nM) when analyzed by FCCS. Modified and reprinted with permission.^126^ Copyright 2015, John Wiley and Sons. (C) Nanomimics from (B) successfully inhibited merozoite invasion and surface‐binding was visualized by fluorescence microscopy (left, one merozoite bound to one RBC) and electron microscopy (right, zoom shows a single polymersome bound on the outer merozoite membrane). Modified and reprinted with permission.^125^ Copyright 2014, American Chemical Society. FCCS, fluorescence cross‐correlation spectroscopy; RBC, red blood cell.

Marques et al. have evaluated side‐on attached heparin on liposomes, either through electrostatic^129^ or covalent attachment (Figure 5A).^123^ The latter was shown to reduce the unwanted anticoagulation property of heparin, which is a benefit over soluble heparin and highlights an advantage of the covalent method.^122^ However, empty heparin‐liposomes with side‐on attached heparin were not more efficient invasion inhibitors than soluble heparin and blood circulation time was relatively short. Thus, the authors shifted their focus to drug delivery, using a combined action of heparin‐liposomes loaded with a conventional antimalarial drug. Electrostatically conjugating heparin in the side‐on configuration on cationic chitosan NPs or dendrimers also caused these NPs to show invasion inhibitory activity against *Plasmodium falciparum*, although again no improved activity was found when compared to soluble heparin.^122^, ^124^ To build membranous NPs with higher physicochemical stability and a more biomimetic presentation of heparin, polymer‐based vesicles (polymersomes) were employed.^125^ A block copolymer was synthesized with end‐on attached heparin serving as the hydrophilic block, which was subsequently mixed with a vesicle‐forming copolymer. These NPs were termed nanomimics due to their nanoscale host cell membrane mimetic structure. Nanomimics were over 100‐fold more efficient than soluble heparin in inhibiting *P. falciparum* merozoite invasion of RBCs.

The mechanism of action of the nanomimic system against merozoites was subsequently analyzed in detail. Giant, micron‐scale polymersomes built with the same nanomimic materials revealed that the end‐on attached heparin successfully bound fluorescently labeled *P. falciparum* (clone 3D7) major surface protein 1–42 (*Pf*MSP1_42_, Figure 5B).^130^ This parasite ligand was previously identified to be responsible for the heparin interaction.^118^ However, there are many additional heparin‐binding ligands present on merozoites,^131^, ^132^, ^133^ providing a multitude of binding‐partners for these NPs. Due to the high prevalence of *Pf*MSP1 on the merozoite surface, this ligand remains the most likely binding partner. Indeed, strong interaction of *Pf*MSP1_42_ with the nanoscale version (nanomimics) was revealed by fluorescence cross‐correlation spectroscopy (*K*_*D*_ ∼12 nM, Figure 5B).^126^ The invasion inhibition mechanism was confirmed by fluorescence and electron microscopy that visualized NP accumulation on the surface of egressed merozoites (Figure 5C). Interestingly, the soft nanomimics slightly adapted their structure to the merozoite surface by flattening out, which was likely caused by the strong multivalent interactions with *Pf*MSP1_42_. This provides evidence that NPs that can adapt to the pathogen surface are more potent parasite inhibitors. This is in agreement with the viral inhibition studies that showed better performance of soft versus rigid NPs.^53^, ^60^ However, in vivo parasite invasion inhibition has not yet been demonstrated with any of the described heparinized vesicle structures.

**FIGURE 6:**
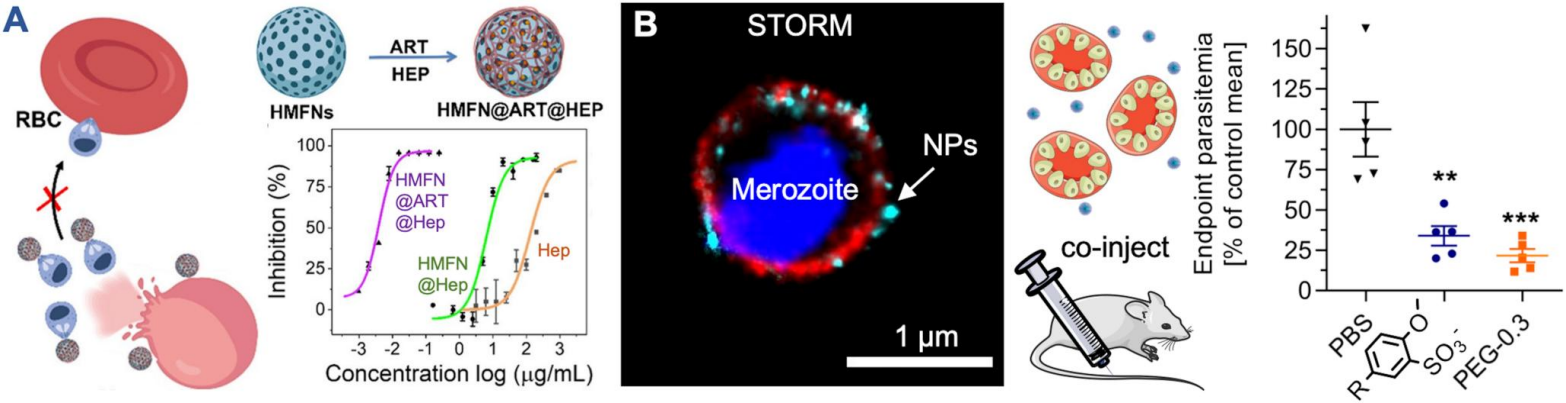
Inorganic and polymeric nanoparticles for *Plasmodium* merozoite inhibition. (A) Schematic and corresponding data showing the process of merozoite inhibition after egress using HMFN@Hep and a combined effect when incorporating an additional antimalarial drug (ART = artemisinin). Modified and reprinted with permission.^127^ Copyright 2021, American Chemical Society. (B) STORM image of a single inhibited *Plasmodium falciparum* merozoite (*Pf*MSP1, red; nucleus, blue) using polymeric NPs (cyan). Co‐injection of late‐stage iRBCs (*Plasmodium berghei*) with polymeric NPs and polymer‐lipid NPs (PEG‐0.3) revealed the functionality of these inhibitors in vivo. **p < 0.01, ***p < 0.001. Modified and reprinted under terms of the CC‐BY license.^55^ Copyright 2022, The Authors, published by American Chemical Society. NPs, nanoparticles; STORM, stochastic optical reconstruction microscopy.

Inorganic hollow mesoporous ferrite NPs were coated with heparin (HMFN@Hep) to serve as an alternative NP system for application against malaria (Figure 6A).^127^ Merozoite invasion inhibition with HMFN@Hep was more efficacious than soluble heparin, again likely due to multivalent interactions of NPs with the merozoites. However, the side‐on configuration of heparin on HMFN@Hep yielded less efficient NPs^127^ versus the end‐on heparinized nanomimics (Table 2).^125^ Since these HMFN@Hep were also found to interact with late‐stage infected RBCs, the authors combined the heparin invasion inhibitory effect with drug release of a conventional antimalarial (artemisinin) from the NP. This combination achieved a higher activity of HMFN@ART@Hep versus HMFN@ART. It was speculated that increasing the local drug concentration improved antimalarial efficacy, although this has not yet been demonstrated in vivo. In a similar application, artesunate was directly coupled to heparin to form NPs without another carrier material, which allowed for controlled drug release and slightly improved drug circulation time.^134^

All these previous anti‐parasitic nanoscale inhibitors included heparin itself as a building block, which was often associated with the unsolved challenges of low efficacy, danger of potential unwanted anticoagulation activity (e.g., after NP disassembly), short blood circulation half‐lives, and applicability in vivo has not yet been tested.^123^ To tackle these challenges, Najer et al. have recently designed HS/SA‐mimetic polymer and lipid‐polymer NPs that were identified as a viable alternative to heparinized NPs.^55^ These NPs were designed with pendant carboxylic/sulfonic acid containing polymers to achieve potent merozoite invasion inhibition (Table 2, Figure 6B). The absence of heparin in the designs reduced the potential for unwanted anticoagulation activity. This was confirmed by antifactor Xa activity tests revealing a negligible activity for these synthetic polymeric NPs (<1% vs. heparin). Various versions of the NPs (carboxylates/sulfonates) potently inhibited *P. falciparum* strains with SA‐dependent (W2mef) and SA‐independent (3D7, D10) invasion mechanisms and even a different species, *P. knowlesi* (A1‐H.1) cultured in human RBCs. Stochastic optical reconstruction microscopy images revealed binding of the NPs to the *Pf*MSP1 outer layer on merozoites (Figure 6B). Interestingly, inhibition of strain W2mef was most efficient, as these parasites require both HS and SA to enter host RBCs. The best polymeric NP inhibitors for parasites included methoxybenzenesulfonate (AMBS) moieties, which were less efficient against viral entry (HSV‐2, Figure 3D). The virucidal AuNPs (MUS/OT)^43^ tested against *P. falciparum* parasites showed much lower inhibition potential than the virustatic polymer NPs (AMSA/AMBS). Hence, virus and malaria parasite inhibition require slightly different designs for optimal activity. A biocidal mechanism might not be required for *Plasmodium* spp. as merozoites lose their invasive potential within a few minutes.^135^, ^136^ The non‐toxic and potent AMBS‐modified copolymer was taken forward and co‐assembled with lipids, including a PEGylated lipid to yield polymer‐lipid nanomimics. This produced the best inhibitor of this series. The incorporation of PEG also increased circulation time when tested in the zebrafish embryo model. Most importantly, these NPs were the first to show malaria parasite invasion inhibition in vivo (*Plasmodium berghei*, Figure 6B). Nevertheless, further in vivo optimization will be required to find optimal dosing and timing of injection, as well as studying subsequent immune responses. The vision for these NPs is to block merozoites in the blood stream and then deliver these complexes to immune cells. This system is desired to induce a stronger immune response to extracellular merozoites for better protection from subsequent infections. The recent finding that these NPs also inhibit and reverse sequestration, that is, binding of *P. falciparum* infected RBCs to endothelial cells (e.g., via chondroitin sulfate A or intercellular adhesion molecule 1 host receptors), which is a hallmark of severe malaria forms, encourages further development of this system.^137^

### 3.2 Invasion inhibition of other protozoan parasites with NPs

In terms of inhibiting other protozoan parasites from entering their respective host cells, only very few NP studies have been performed to date. The previous focus was on drug delivery applications and on inherent toxic effects on parasites or host stimulatory mechanisms predominantly provided by metal‐based NPs.^28^, ^29^, ^30^, ^31^, ^32^, ^33^, ^34^, ^35^, ^36^ Stearylamine‐liposomes were active against a range of parasites as summarized recently, but due to their cationic nature, potential interaction with host cells has to be taken into account.^138^ Here, some examples of surface‐binding NPs and information from tests with soluble heparin are combined. These examples inform on considerations necessary for designing HS/SA containing/mimetic NPs that target protozoan parasites other than *Plasmodium* spp.

**FIGURE 7:**
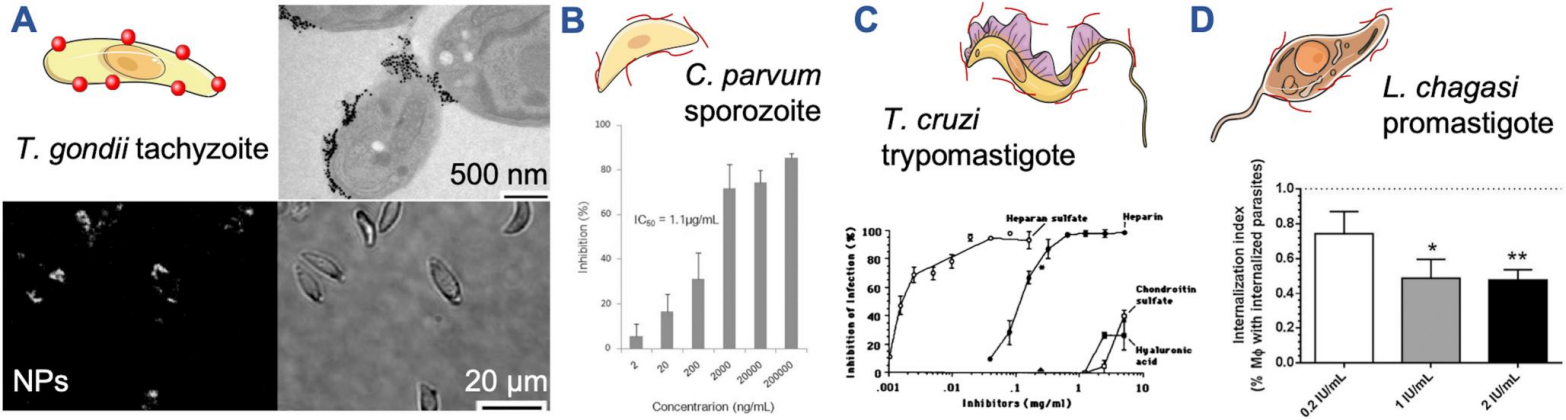
Invasion inhibition of various protozoan parasites. (A) *Toxoplasma gondii* tachyzoites were inhibited by surface‐binding AuNPs functionalized with an anti‐*T. gondii* antibody, as demonstrated by electron microscopy (top) and fluorescence imaging (bottom). Modified and reprinted with permission.^139^ Copyright 2009, John Wiley and Sons. (B) *Cryptosporidium parvum* sporozoite host cell entry was inhibited with soluble heparin. Modified and reprinted under terms of the CC‐BY license.^18^ Copyright 2015, The Authors, published by Springer Nature. (C) *Trypanosoma cruzi* trypomastigotes were hindered from entering host cells by treatment with HS and heparin, while chondroitin sulfate and hyaluronic acid were inactive. Modified and reprinted with permission.^140^ Copyright 1991, Elsevier. (D) Heparin treatment of *Leishmania chagasi* promastigotes reduced macrophage infection. *p < 0.05, **p < 0.01. Modified and reprinted with permission.^141^ Copyright 2015, Elsevier. All schematics were modified from Servier Medical Art website CC‐BY.

Invasion inhibitory NPs were previously designed against *T. gondii* host cell invasion but using a specific interaction (Figure 7A).^139^ 20 nm AuNPs functionalized with an anti‐*T. gondii* antibody were shown to bind to *T. gondii* tachyzoites using fluorescence and electron microscopy. Surface binding inhibited invasion successfully; however, the potency was not improved over soluble antibody. Others also investigated a more generic effect when preincubating tachyzoites with AgNPs.^142^ Adherence and infection was lowered, but the mechanism remains unclear. NP‐mediated tachyzoite deformation and intracellular reactive oxygen species production are likely explanations, as found in other AgNP studies.^143^, ^144^ *Toxoplasma gondii* tachyzoites also bind heparin, as demonstrated with heparin‐functionalized NPs, although only soluble heparin was tested as invasion inhibitor.^145^ A 90% reduction in host cell infection was achieved at a concentration of 10 μg/mL heparin, confirming the involvement of HS in *T. gondii* tachyzoite host cell invasion. This encourages the development of HS‐mimetic NPs for toxoplasmosis.

*Cryptosporidium parvum* sporozoite entry into host cells was potently blocked by soluble heparin (Figure 7B).^18^, ^146^ The related polysaccharide fucoidan also revealed invasion inhibitory activity in vitro.^18^ Encouragingly, oral administration of fucoidan in mice prior and after parasite inoculation reduced the number of parasites 5‐fold.^147^ Hence, the theoretical basis for an anti‐cryptosporidial invasion inhibitory NP leveraging the HS interaction is given. However, the administration route, ideally through oral dosing, will be a key consideration because NPs will have to act in the intestine where *C. parvum* replicates.

Current nanotherapeutic strategies against Chagas disease, caused by *T. cruzi*, have also focused on drug delivery.^148^, ^149^, ^150^ Surface interaction of NPs with invasive trypomastigotes is not yet a major avenue being explored. Cationic stearylamine‐liposomes interacted with anionic extracellular *T. cruzi* trypomastigotes, which was leveraged for drug delivery.^148^ However, the short circulation time of stearylamine‐liposomes hinders their application via systemic application. If, alternatively, the NPs enter the host cells after infection, the NPs need to escape the endosome to reach and act against cytosolic *T. cruzi*. Exiting the endosome after NP uptake is a key challenge in the entire NP‐based drug delivery field. Supportive evidence that surface binding alone can reduce the host cell infection by *T. cruzi* trypomastigotes was provided when testing antibodies.^151^ *Trypanosoma cruzi* trypomastigotes and amastigotes were also found to bind heparin/HS, as preincubation with these inhibitors reduced host cell invasion (Figure 7C).^140^, ^152^ This encourages HS‐mimetic nanoscale inhibitor formulation for Chagas disease.

In contrast to the previous examples, a surface‐binding NP strategy might have little effect on protozoan parasites that enter host cells via host cell dominated processes such as phagocytosis. This is the case for macrophage infection with *Leishmania* spp. promastigotes/amastigotes.^153^ Exceptions would be NPs that can directly affect promastigote/amastigote viability. Alternatively, binding drug‐loaded NPs to the parasite surface could allow the release of the loaded drug locally after the phagocytosis of the NP‐parasite complex. These considerations are the reason why most research studies to date have focused on macrophage‐targeted activity of NPs for leishmaniasis (e.g., cytotoxic metal NPs and drug delivery).^30^, ^31^ There is an ongoing debate on the influence of heparin on *Leishmania* spp. host cell infection.^154^ *Leishmania chagasi* promastigote and *Leishmania amazonensis* amastigote infection of macrophages was previously reduced by heparin.^141^, ^155^ However, others have shown that heparin can increase macrophage interaction with *Leishmania donovani* promastigotes.^156^ Heparin in the context of nanomedicine for *Leishmania* was evaluated recently as an inhalable heparin/chitosan NP for drug delivery.^157^ In this example, heparin increased macrophage uptake of drug‐loaded NPs. Hence, it remains to be seen whether HS mimetic polymers or NPs have a therapeutic potential for leishmaniasis.

## 4 CONCLUSIONS AND OUTLOOK

NPs which contain or mimic HS/SA have emerged as promising candidates for the development of broad‐spectrum attachment/entry/invasion inhibitors for various viral and protozoan infections. This concept could provide urgently needed treatment options for these devastating diseases. For NPs that act against viral attachment/entry, a key consideration is the processes that occur after NP‐virus binding.^25^ This was a key learning point from the failure of anionic inhibitors in clinical trials against HIV.^46^ After administration, the NPs will inevitably be diluted over time due to diffusion, fluid flow, and uptake by immune cells. If the NP‐virus interaction is based on a reversible interaction, this dilution could again liberate the infective virus, potentially rendering the inhibition strategy ineffective. This was circumvented recently by formulating HS/SA mimetic virucidal NPs. These virucidal NPs not only bind to viruses but also inactivate them by disrupting the viral structure.^25^, ^43^, ^49^, ^54^, ^57^, ^73^ Compared to viruses, protozoan parasites are much more complex unicellular eukaryotic pathogens with thousands of proteins involved in their life cycles. However, the involvement of the same HS/SA attachment receptors in host cell invasion of various obligate intracellular protozoan parasites was confirmed as summarized herein.^18^, ^118^, ^140^, ^141^, ^145^ Recent demonstration that broad‐spectrum HS/SA‐mimetic NPs are also applicable as malaria parasite invasion inhibitors in vitro and in vivo (Table 2) should further encourage the development of HS/SA‐mimetic NPs for various parasitic diseases.

Despite all these advances, there are several outstanding challenges that must be addressed before these NPs can be translated into medicines against viral and parasitic pathogens. The first challenge concerns both types of pathogens. Most viruses and obligate intracellular parasites enter their host cells very rapidly. Hence, the route and timing of administration will be key for any entry/invasion inhibitory NPs. This represents a key difference to conventional drug delivery via NPs, which often functions by delivering the drug to infected cells. Since intracellular pathogen development is a much longer process than entry/invasion, timing for the administration of drug delivery NPs is less important than for NPs inhibiting entry/invasion. However, targeting extracellular pathogens with NPs also comes with the advantage that the challenges characteristic to drug delivery systems, that is, the need to cross various membranes and releasing the cargo compound in the correct compartment, fall away. To deal with the shorter time window for the application of entry/invasion inhibitory NPs, administration pathways that follow the main infection or distribution routes of the pathogen are likely to be most successful to maximize the number of NPs brought together with the extracellular forms of the pathogens. For example, respiratory viruses such as IAV and SARS‐CoV‐2 are best tackled by administering via the respiratory tract.^19^, ^158^ In contrast, inhibiting most protozoan infections appears to be more applicable through oral dosing or i.v. injection. NP administration for *Cryptosporidium* spp. could follow the oral route as this pathogen replicates in the intestines. Injection via the i.v. route could be appropriate for *Plasmodium* spp. merozoites that exclusively replicate in the bloodstream. Similarly, *T. gondii* tachyzoites also distribute through the blood in the acute stage, and in visceral leishmaniasis spleen and liver are heavily affected,^32^ which is the same destination as for most i.v. applied NPs. However, for diseases most prevalent in low‐ and middle‐income countries, and especially for application in rural settings, i.v. administration is not readily applicable. In these situations, oral dosing would be much more appropriate. However, formulation development to produce stable NPs that are efficiently taken up into the bloodstream after oral administration is still an area for further development.

Another challenge to the translation of these inhibitory NPs, applicable to both viruses and parasites, is the locally encountered conditions after administration, such as highly proteinaceous environments or low pH. These complex environments impact NP stability and potency in vivo. The importance of protein corona formation on nanoscale drug delivery systems has only been realized recently.^70^, ^71^ This protein corona formation could particularly impact surface‐active NPs as discussed herein, which must be studied in more detail. Other important properties that must always be investigated are NP biocompatibility, biodistribution, elimination and degradation. This is especially important for NP designs that were found to have inherent toxic effects on pathogens as they might also negatively affect host cells, which has been a known issue for some types of metal‐based NPs, for example.^77^

With respect to virus inactivation or elimination after NP binding, other strategies could be evaluated further. Indirect viral inactivation could potentially be incorporated through mimicking natural processes. For example, mechanisms of action of natural or vaccine‐induced NAbs could serve as an inspiration. These specific inhibitors can either block viral entry or influence post‐entry processes after NAb‐virus binding.^159^ This binding is also reversible, but slowing down viral entry can cause irreversible virus damage through other mechanisms: (i) uptake of NAb‐virion complexes by immune cells via Fc receptors leads to endocytosis and lysosomal degradation, (ii) NAbs force cell‐membrane fusing viruses into the endocytic pathway, or (iii) NAbs delay viral endolysosomal membrane fusion, which increases the residence time of these viruses in this degrading compartment.^159^ Therefore, NP designs with a reversible binding mechanism, similar to NAbs, but with “NAb‐like” mechanisms to trigger the viral damaging mechanisms described above (i–iii) could be interesting future avenues of research. A recent example used 2D nanosheets to capture SARS‐CoV‐2 which led to macrophage uptake and subsequent lysosomal degradation of the virus.^115^ Hence, NPs with high virustatic potency as highlighted throughout the review could serve as a promising base platform for the development of such approaches. However, testing these mechanisms will require more sophisticated in vitro co‐culture and in vivo analysis. Although there are several hurdles, including translational challenges, left to solve before broad‐spectrum antiviral NPs are brought to the market, they have clear potential, which should be explored further to eventually tackle ongoing and future viral diseases.^25^, ^160^ As one advantage, the broad‐spectrum design of therapeutic NPs would allow immediate testing and approval if found effective against an upcoming viral disease, which could provide an urgently needed tool to bridge the time needed for developing, testing, approving, manufacturing, and rolling out efficacious vaccines.

Moving from HS/SA‐mimetic NPs to treat viral diseases to using these NPs to inhibit infection by obligate intracellular protozoan parasites brings a different set of challenges to consider. The unavailability of suitable in vitro models for the propagation of several protozoan pathogens for inhibitor testing is one obstacle for some of these diseases, for example, *Cryptosporidium* spp. Particularly, more realistic models employing “organoids‐on‐a‐chip” technology, such as “mini‐intestines” for *C. parvum* propagation,^161^ could have a big impact on the development of novel treatments. High strain variability, antigenic diversity, antigenic variation, and surface shedding of invasion ligands are other parasite specific challenges for any inhibitor targeting the parasite surface. These parasite properties highlight the difficulty in designing inhibitors based on specific interactions, such as vaccine‐induced NAbs, and partly explain the lack of efficacious vaccines for most parasites. Broad‐spectrum approaches could address this issue because they can simultaneously target several different parasite ligands and functions against various parasite strains and species. The parasites' more complex structures and life cycles are also associated with some advantages for NP inhibitor design. For example, *Plasmodium* extracellular forms (merozoites) are extremely short‐lived extracellularly (invasive only for a few minutes),^135^, ^136^ which could make a biocidal NP inhibition mechanism unnecessary. However, this requires more in vivo evaluation to confirm.

Pathogen‐binding NPs could potentially also serve other purposes besides simple attachment/invasion inhibition due to their multifunctionality. NPs can be equipped with further targeting moieties and loaded with immunomodulatory molecules or molecular drugs. For example, delivering NP‐pathogen complexes to immune cells could represent a strategy to potentially build up stronger immunity to protect against future infections. In addition, the incorporation of immune cell targeting ligands and/or immunomodulatory compounds in the NP design could provide means to modulate this immune response. However, such applications remain to be demonstrated in practice. If surface‐binding of NPs proves to be inefficient in inhibiting some of the various protozoan parasites discussed herein, combinations with molecular drugs should be evaluated further. Drug‐loaded NPs bound to the parasite surface might be dragged into the host cell during the invasion, which could increase the local drug concentration after release from the NPs.

In summary, nanomedical approaches utilizing pathogen‐binding NPs against various viral and protozoan parasite infections show promise to be established as alternative therapeutic tools. This review aims to inspire more exploratory and translational research into nanotherapeutics against these impactful diseases. It is envisioned that the field of pathogen‐binding NPs could eventually provide alternative opportunities for controlling various infectious diseases important in global health.

## AUTHOR CONTRIBUTIONS

Adrian Najer prepared the figures and wrote the manuscript.

## CONFLICT OF INTEREST STATEMENT

The author declares that there are no conflicts of interest.
